# Correction: Phosphorescent [3 + 2 + 1] coordinated Ir(iii) cyano complexes for achieving efficient phosphors and their application in OLED devices

**DOI:** 10.1039/d4sc90079k

**Published:** 2024-06-21

**Authors:** Yuan Wu, Chen Yang, Jie Liu, Meng Zhang, Weiqiang Liu, Wansi Li, Chengcheng Wu, Gang Cheng, Qingdan Yang, Guodan Wei, Chi-Ming Che

**Affiliations:** a PURI Materials 6F, Block A, Jiazhaoye Xindong Kechuang Park, 71st Zone Xindong, Baoan District Shenzhen 518133 China david_yang@purimat.com; b School of Chemical Engineering and Light Industry, Guangdong University of Technology Guangzhou 510006 China qdyang@gdut.edu.cn; c State Key Laboratory for Mechanical Behavior of Materials, Xi’an Jiaotong University Xi’an 710049 China; d Tsinghua-Berkeley Shenzhen Institute (TBSI), Tsinghua Shenzhen International Graduate School, Tsinghua University Shenzhen 518055 China weiguodan@sz.tsinghua.edu.cn; e State Key Laboratory of Synthetic Chemistry, HKU-CAS Joint Laboratory on New Materials, Department of Chemistry, The University of Hong Kong Pokfulam Road Hong Kong SAR China ggcheng@hku.hk; f HKU Shenzhen Institute of Research and Innovation Shenzhen 518053 China

## Abstract

Correction for ‘Phosphorescent [3 + 2 + 1] coordinated Ir(iii) cyano complexes for achieving efficient phosphors and their application in OLED devices’ by Yuan Wu *et al.*, *Chem. Sci.*, 2021, **12**, 10165–10178, https://doi.org/10.1039/D1SC01426A.

The authors regret that funding details were incorrect in the acknowledgements section of the original article. The corrected acknowledgements section for this article is shown below.

## Acknowledgements

W. Y., C. Y. and C.-C. Wu acknowledge Shenzhen PURI Materials Technologies, Co., Ltd. and colleagues for their support. This work was supported by the Guangdong Major Project of Basic and Applied Basic Research (2019B030302009), National Natural Science Foundation of China (NSFC No. 52003059), the Guangzhou Science and Technology Program (No. 202002030397), the Shenzhen Science and Technology Innovation Committee (JCYJ20190809172615277), and the Science and Technology Planning Project of Shenzhen Municipality (JCYJ20200109144614514).

The Royal Society of Chemistry apologises for these errors and any consequent inconvenience to authors and readers.

